# Increased frequency and intensity of complicated migraine sans migraine after third BNT162b2 dose

**DOI:** 10.1016/j.clinsp.2023.100315

**Published:** 2024-03-12

**Authors:** Larissa M. Bombardi, Antonio-Carlos Guimaraes de Almeida, Josef Finsterer, Fulvio Alexandre Scorza

**Affiliations:** a Disciplina de Neurociência, Universiade Federal de São Paulo/Escola Paulista de Medicinal (UNIFESP/EPM), São Paulo, SP, Brazil; b Centro de Neurociências e Saúde da Mulher “Professor Geraldo Rodrigues de Lima”, Escola Paulista de Medicina/Universidad Federal de São Paulo (EPM/UNIFESP), São Paulo, SP, Brazil; c Neurology & Neurophysiology Center, Vienna, Austria

## Commentary

There is increasing evidence from real-world data that anti-SARS-CoV-2 vaccines are not free of side effects and therefore are not safe for everyone [1]. There are even side effects that can be fatal [2]. Whether patients with pre-existing conditions generally experience aggravation or deterioration of their pre-vaccination condition is unclear, but there is evidence that pre-morbid conditions may worsen following anti-SARS-CoV-2 vaccinations [3], as in the following case.

The patient is a 28-year-old female with complicated migraines without migraines with three attacks in a 12-year period. The migraine presented with unilateral blurred vision followed by a typical ipsilateral scintillation scotoma for 30 min, followed by dysarthria and ipsilateral sensory disturbances of the ipsilateral upper extremity, lasting an additional 10 min. The attacks were triggered by glare and hormonal imbalances.

After receiving the third dose of the BNT162b2 vaccine, the index patient experienced an increase in the frequency and intensity of migraine attacks. The first and second BNT162b2 doses had no effect on migraine frequency. Since the third dose, the patient suffered a total of six migraine attacks within five months (Table 1). The first, like the previous ones, manifested itself in the left eye, but additionally with bifrontal headache. The second attack manifested as before the third vaccination. The third attack occurred just before menses and presented with diplopia for 5‒10 min, scintillation scotoma, and bifrontal headache, but without sensory disturbances (Table 1). The fourth attack began with diplopia for 5‒10 min, followed by right-sided scotoma mild dysarthria, and right-sided sensory disturbances, including the face. The fifth attack was of the usual kind. The sixth attack was as usual but without sensory disturbances. The clinical examination four days after the 6th attack revealed only sore neck muscles. Cerebral MRI, extensive blood tests, ophthalmologic examination, and EEG were non-informative. Tizanidine (2 mg) and zolmitriptan (2.5 mg) were prescribed with beneficial effects.

**Table 1:** Clinical manifestations and frequency of migraine attacks over the last 12 years and after receiving the third BPV dose.

Date	Side	BV	SS	DA	ISD	HA	DV
2010	NR	X	X	X	X	‒	‒
2016	NR	X	X	X	X	‒	‒
2/2020	NR	X	X	X	X	‒	‒
12/21	Third dose of BPV						
29.1.22	Left	X	X	X	X	X	‒
17.2.22	Left	X	X	X	X	‒	‒
21.4.22	Left	‒	X	‒	‒	X	X
27.4.22	Right	‒	X	X	X	-	X
1.5.22	Right	X	X	X	X	‒	‒
7.5.22	nr	X	X	X	‒	‒	‒

BV, Blurred Vision; DA, Dysarthria; DV, Double Vision, SS, Scintillating Scotoma; ISD, Ipsilateral Sensory Disturbance; HA, Headache; NR, Not Reported.

This case shows that SARS-CoV-2 vaccinations can increase the frequency and intensity of attacks of a complicated non-migraine migraine. Other causative factors such as stress, infections, drugs, sleep disturbance, weather changes, or sensory stimuli were excluded. Previous reports have shown that SARS-CoV-2 vaccinations can trigger de novo migraine attacks or increase the frequency and intensity of migraine attacks. According to an evaluation of the WHO pharmacovigilance database (VigiBase), each of the anti-SARS-CoV-2 vaccines can trigger migraine attacks [4]. In a case series of eight patients, four developed migraines 1‒7d after the CoronaVac vaccine (Sinovac) [5]. Patients with migraine showed large areas of hypoperfusion and small areas of hyperperfusion on HMPAO-SPECT [5]. In a 37-year-old female with a history of migraines, the second dose of BNT162b2 vaccine induced status migrainosus [6]. In a 24-year-old female the CoronaVac vaccine induced a prolonged motor aura, manifested as left hemiparesis and left hypoesthesia [7]. HMPAO-SPECT showed a significant hypoperfusion of the right hemisphere [7].

The pathophysiology of triggering de novo migraine attacks or worsening of pre-existing migraines by anti-SARS-CoV-2 vaccination is unclear, but it can be speculated that the immune response induced by vaccination also affects endothelial or vascular smooth muscle cells and thereby impairs vascular contractility. It is also conceivable that the vaccine triggers the release of vasoactive substances, thereby altering the physiological response to conditions that alter vascular tone. It is also conceivable that antibodies induced by the vaccine alter the responsiveness of the cerebral arteries. In any case, the described change in the type and intensity of the migraine must be classified as Long Post-COVID Vaccination Syndrome (LPCVS) [8].

This case shows that SARS-CoV-2 vaccinations can increase the frequency and intensity of pre-existing complicated migraine sans migraine. The authors suspect that the immune response against the vaccine also affects endothelial or vascular smooth muscle cells, thereby impairing vascular contractility.

## Ethics approval

Was in accordance with ethical guidelines. The study was approved by the institutional review board.

## Consent to participate

Was obtained from the patient.

## Consent for publication

Was obtained from the patient.

## Availability of data

All data are available from the corresponding author.

## Code availability

Not applicable.

## Author's contribution

Design, literature search, discussion, first draft, critical comments, and final approval.

## Funding

No funding was received.

## Declaration of Competing Interest

The author declares that the research was conducted in the absence of any commercial or financial relationships that could be construed as a potential conflict of interest.
